# Concordance et apports de l'hystérosalpingographie et de la cœlioscopie dans l'exploration tubaire et pelvienne en cas d'infertilité

**DOI:** 10.11604/pamj.2014.17.126.3567

**Published:** 2014-02-21

**Authors:** Mehdi Kehila, Rim Ben Hmid, Sonia Ben Khedher, Sami Mahjoub, Mohamed Bedis Channoufi

**Affiliations:** 1 Faculté de Médecine de Tunis, Service C du Centre de Maternité et de Néonatologie de Tunis, Tunis, Tunisie

**Keywords:** Hystérosalpingographie, cœlioscopie, infertilité, adhérences péritonéales, perméabilité tubaire, hysterosalpingography, laparoscopy, infertility, peritoneal adhesions, tubal patency

## Abstract

**Introduction:**

La coelioscopie et l'hystérosalpingographie sont les deux procédures usuellement admises pour l'exploration tubaire et pelvienne en cas d'infertilité. Les objectifs de ce travail etaient de comparer les données de l'Hystérosalpingographie à celles de la coelioscopie chez des patientes explorées pour infertilité et voir l'apport de l'une par rapport à l'autre.

**Méthodes:**

Etude prospective menée au service C du centre de maternité et de néonatologie de Tunis, s’étendant sur 15 mois, colligeant 120 patientes suivies pour infertilité ayant bénéficié d'une HSG suivie d'une coelioscopie.

**Résultats:**

L’âge moyen de nos patientes était de 35,3 ans. L'infertilité était primaire dans 56,7% des cas et secondaire dans 43,7% des cas. Sa durée moyenne était de 48,9 mois. Le test statistique de concordance Kappa entre les 2 examens était de 0.42 pour les obstructions tubaires en général, de 0.48 pour les obstructions tubaires proximales et de 0.53 pour les obstructions tubaires distales indiquant une concordance modérée dans tous les cas. La coelioscopie a permis d'objectiver en plus une endométriose pelvienne dans 7% des cas, des adhérences pelviennes dans 33% des cas et des trompes perméables mais d'aspect pathologique dans 20% des cas.

**Conclusion:**

Il existe un intérêt d′associer, chaque fois que c′est possible, l'HSG et la coelioscopie dans l'exploration du pelvis féminin dans le cadre de l'infertilité. Aucun de ces deux examens n'est parfait. Leurs résultats sont complémentaires.

## Introduction

L'exploration d'un couple infertile est un processus complexe comportant plusieurs volets: anatomique, fonctionnel et psychologique. L'exploration du versant féminin nécessite des examens complémentaires morphologiques et biologiques. La coelioscopie et l'hystérosalpingographie (HSG) sont les deux procédures utilisées pour l'exploration tubaire et pelvienne. La coelioscopie est considérée par la plupart des auteurs comme le « gold standard » dans cette indication [1]. Nous nous sommes proposé dans ce travail de comparer les données de l'HSG à celles de la coelioscopie chez des patientes explorées pour infertilité et ceci afin de voir le degré de concordance entre les 2 examens et l’éventuel apport de l'un par rapport à l'autre.

## Méthodes

Nous avons mené une étude prospective au service C du centre de maternité et néonatologie de Tunis, s’étendant sur 12 mois, allant du premier janvier jusqu'au 31 Décembre 2013, colligeant 120 patientes suivies pour infertilité chez qui a été pratiquée une HSG suivie d'une coelioscopie. Durant la période d’étude, nous avons proposé à toute les patientes explorées pour infertilité et acceptant de participer à l’étude, d'associer de façon systématique une HSG suivie d'une coelioscopie. Au cours de la laparoscopie, La perméabilité tubaire a été vérifiée dans tous les cas par une épreuve au bleu de méthylène. Toutes les patientes participant à l’étude ont donné leur consentement éclairé par écrit.

Etait inclue dans notre étude toute patiente explorée pour infertilité dont le spermogramme du conjoint était compatible avec une insémination intra-utérine. Les critères d'exclusion étaient un intervalle de plus de 3 mois entre les 2 examens, les patientes à haut risque anesthésique (intubation prévue difficile, toute patiente présentant un problème respiratoire ou cardiaque), ainsi que celles à haut risque chirurgical (indice de masse corporelle - 30, abdomen bicicatriciel et plus). Les données recueillies ont été analysées à l'aide du logiciel SPSS (Version 12.0.1; SPSS Inc., Chicago, IL, USA). La statistique Kappa (K) a été utilisée pour préciser la concordance entre les résultats de l'HSG et ceux de la coelioscopie.

**Clearance éthique:** Cette étude a été approuvée par la Commission d′éthique du centre de maternité et de néonatologie de Tunis.

## Résultats

Les principales caractéristiques épidémiologiques de nos patientes sont résumées dans le Tableau 1. Aucune complication sérieuse n'a été notée pendant la période d’étude.

**Tableau 1:** Caractéristiques épidémiologiques des patientes

Age	35,3 ans (22-45)
Type de l'infertilité	
Primaire	68 (56,7%)
Secondaire	52 (43,7%)
Durée moyenne de l'infertilité (mois)	48,9 (9-180)
Antécédent de chirurgie abdomino-pelviennes	
Appendicectomie	4 (4,3%)
Myomectomie	8 (8,6%)
Plastie tubaire	4 (4,3%)
Kystectomie ovarienne	4 (4,3%)
Salpingectomie	2 (2,15%)
Césarienne	4 (4,3%)
**Autres antécédents gynéco-obstétricaux**	
Assistance médicale à la procréation	10 (8,3%)
Contraception par dispositif intra-utérin	10 (8,3%)
Infection génitale haute	2 (1,7%)
Grossesse extra-utérine	4 (3,4%)
Fausse couche sponanée	14 (11,7%)
Manaeuvres endo-utérines	8 (6,7%)

**Figure 1:**
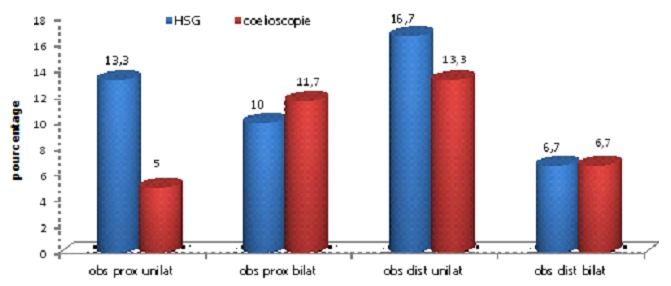
Données globales de l'hystérosalpingographie et de la caelioscopie

Les données globales de l'HSG et de la coelioscopie sont résumées dans la Figure 1. Nous avons comparé les résultats de l'HSG et ceux de la coelioscopie dans l’évaluation: de la perméabilité et de l’état tubaire en général, des obstructions tubaires proximales, des obstructions tubaires distales.

La concordance HSG-coelioscopie dans la perméabilité tubaire en général est résumée dans le Tableau 2. L'HSG concordait avec la coelioscopie pour dire qu′il existait une obstruction tubaire dans 69,3% des cas (54/78). Dans 30,7% des cas (24/78) l′HSG montrait des trompes perméables alors qu′elles étaient jugées occluses à la coelioscopie. La corrélation HSG-coelioscopie dans les obstructions tubaires proximales est représentée dans le Tableau 3. Les résultats de l'HSG et ceux de la coelioscopie concernant les obstructions tubaires distales sont résumées dans le Tableau 4.

**Tableau 2:** Concordance HSG-cœlioscopie dans la perméabilité tubaire en général

HSG	Cœlioscopie		Total
	Trompes occluses	Trompes perméables	
Trompes occluses	54 (45%)	10 (8,3%)	64 (53,4%)
Perméables	24 (20%)	32 (26,7%)	56 (46,6%)
Total	78 (65%)	42 (35%)	120 (100%)

K= 0, 42

**Tableau 3:** Concordance HSG-cœlioscopie dans les obstructions tubaires proximales

HSG	Cœlioscopie		Total
	Pas d'obstruction proximale	obstruction proximale	
Pas d'obstruction proximale	86 (71,7%)	4 (3,3%)	90 (75%)
obstruction proximale	16 (13,3%)	14 (11,7%)	30 (25%)
Total	102 (85%)	18 (15%)	120 (100%)

K= 0,48

**Tableau 4:** Concordance HSG-cœlioscopie dans les obstructions tubaires distales

HSG	Caelioscopie		Total
	Pas d'obstruction Distale	Obstruction distale	
Pas d'obstruction Distale	82 (68,3%)	8 (6,7%)	90 (75%)
obstruction distale	12 (10%)	18 (15%)	30 (25%)
Total	94 (78,3%)	26 (21,7%)	120 (100%)

K= 0,53

En plus de l’évaluation de la perméabilité tubaire, la coelioscopie a permis de mettre en évidence: des adhérences pelviennes non détectées à l'HSG chez 40 patientes (33.4%). Les annexes étaient concernées par ses adhérences dans 16 cas (13%). La sensibilité de l'HSG dans la détection des adhérences pelvi-péritonéales était de 9,5%, une endométriose pelvienne non suspectée à l'HSG dans 7% des cas (8 patientes), des trompes perméables mais d'aspect pathologique (non suspecté à l'HSG) dans 20% des cas (24 patientes).

## Discussion

L'exploration du tractus génital féminin est un des éléments essentiels du bilan d'infertilité. L'HSG, qui est une technique relativement simple, est le plus souvent réalisée en première intention pour évaluer l′anatomie de l′utérus et la perméabilité tubaire [2]. La coelioscopie permet une visualisation directe des trompes, de l'utérus et du pelvis. Elle a un intérêt diagnostic et éventuellement thérapeutique. Elle est considérée par la plupart des auteurs comme le « gold standard » dans l'exploration du pelvis en cas d'infertilité [1]. Cependant, il s'agit d'un examen invasif non dénué de complications [3] et qui nécessite une anesthésie générale. La question qui se pose pour le clinicien est le degré de corrélation entre les données respectives de l'HSG et de la coelioscopie ainsi que l'apport de chacun des deux examens.

Lors de la conception de notre étude, nous étions persuadés comme beaucoup d'autres auteurs [1, 4, 5] que la coelioscopie était l'examen de référence dans l’évaluation tubaire en cas d'infertilité. Ces auteurs, dans leurs séries, partent de cette hypothèse pour calculer la sensibilité, la spécificité, la valeur prédictive positive (VPP) et la valeur prédictive négative (VPN) de l'HSG [4, 5–8]. Ces statistiques, pour évaluer la fiabilité de l'HSG, sont calculées en se basant sur le fait que la coelioscopie a toujours raison. Toutefois, nous constatons que dans plus d'un tiers des cas, des trompes perméables à l'HSG se révèlent occluses à la coelioscopie (35% dans la série de Swart [5]; 30,7% dans notre série). Notamment, dans notre série, nous avons noté 22,8% d'obstructions tubaires proximales à la coelioscopie malgré une HSG normale. Cette constatation nous a poussés à relire les clichés d'HSG en question. Les trompes étaient bel et bien injectées avec un passage péritonéal visible contrastant avec une coelioscopie concluant à une obstruction proximale bilatérale. Il ne s'agit probablement pas dans ces cas d'un manque de sensibilité de l'HSG mais plutôt d'un faux positif de la coelioscopie. Ces fausses obstructions tubaires peuvent être dues à un problème technique lors de la coelioscopie comme une fuite vaginale du colorant, une pression d'injection insuffisante, une quantité insuffisante ou une anesthésie insuffisante entrainant un spasme tubaire. Actuellement, après avoir analysé les résultats de cette étude, en cas de trompes perméables à l'HSG avec une obstruction proximale bilatérale à la coelioscopie, après vérification des différents détails techniques, nous demandons aux anesthésistes d'approfondir l'anesthésie avant de réinjecter le bleu. Cet artifice nous a permis d'objectiver un passage tubaire 3 fois sur 4.

Il nous parait donc évident que la coelioscopie ne peut être prise comme référence pour calculer la sensibilité et la spécificité de l'HSG. Plutôt alors que d'essayer de juger de la fiabilité de l'HSG, il parait plus judicieux d’évaluer la corrélation entre les 2 examens tout en essayant de comprendre les forces et les failles de chacun.

### Concordance HSG-Coelioscopie en cas d'obstruction tubaire proximale

Ce paramètre est intéressant à étudier. En effet une bonne fiabilité de l'HSG rendrait la coelioscopie inutile et justifierais plutôt une salpingographie sélective [8] ou un passage en fécondation in vitro. Les données de la littérature ainsi que notre étude [9] sont en faveur d'une corrélation modérée entre l'HSG et la coelioscopie dans la détection des obstructions proximales (dans notre étude K = 0.48). Dans la série de Mol et al [9], en cas d'obstruction proximale à l'HSG, 40% des coelioscopies ont montré des trompes perméables. L'existence d'une occlusion tubaire proximale à l'HSG justifie donc la réalisation d'une coelioscopie afin d'infirmer ou de confirmer le diagnostic. La présence de faux positifs à l'HSG dans ce cas parait admise et peut être expliquée par les spasmes en réaction à la douleur et les bouchons muqueux [10]. Certaines mesures permettent la diminution du taux de faux positifs de l'HSG tels que l'utilisation des antalgiques, bien rassurer la patiente, bien tirer sur le col pour diminuer une éventuelle anté ou rétroversion et bien sure une bonne interprétation de l'HSG.

### Concordance HSG-c'lioscopie pour l'obstruction tubaire distale

Les obstructions tubaires distales sont accessibles à des gestes thérapeutiques chirurgicaux [10]. Ce diagnostic justifie la pratique d'une c'lioscopie à visée thérapeutique permettant une amélioration de la fertilité spontanée et évite le passage en FIV pour certaines patientes [11]. Cependant peu d’études se sont intéressées à la fiabilité de l'HSG dans le diagnostic des obstructions tubaires distales. Dans notre étude, les 2 examens étaient en accord concernant la perméabilité tubaire distale dans 83,3% des cas avec un test de Kappa à 0.53 indiquant une corrélation modérée entre les 2 examens. En fait, la difficulté dans ce cas, est surtout de différentier à l'HSG une perméabilité tubaire bilatérale d'une obstruction distale unilatérale. En effet, la vision de la trompe injectée jusqu’à sa partie distale associée au brassage péritonéal provenant de la trompe perméable peut facilement prêter à confusion avec une perméabilité bilatérale.

### Fiabilité de l'HSG dans le diagnostic des adhérences pelviennes

La relation entre les adhérences pelviennes et l'infertilité féminine est admise. Sa prévalence chez les femmes infertiles est estimée entre 10 et 23% [12]. La fiabilité de l'HSG dans l’évaluation péritonéale est loin d’être absolue. La plupart des études plaident en faveur de la nette supériorité de la coelioscopie dans cette indication [13]. Nous pensons que, donnant une vision directe du pelvis, la coelioscopie peut être considérée comme l'examen de référence pour la détection des adhérences pelviennes. Dans notre série, en considérant la coelioscopie comme le « Gold standard » dans cette indication, la sensibilité de l'HSG dans la détection des adhérences pelvi-péritonéales était très faible (9,5%).

### Fiabilité de l'HSG dans le diagnostic de l'endométriose pelvienne

L'endométriose pelvienne est la principale pathologie à l'origine des discussions sur la pratique systématique d'une coelioscopie dans le cadre du bilan d'infertilité. Sa prévalence chez une population de femmes infertiles est estimée entre 20 et 68,0% (2,5 à 3,3% de la population générale) [14]. Capelo et al [15] trouvent, dans 50% des cas, une endométriose pelvienne lors de la réalisation de coelioscopie chez des patientes suivies pour une infertilité étiquetée inexpliquée. Dans notre série une endométriose pelvienne a été découverte chez 7% des patientes (non suspectées à l'HSG). Jusqu’à nos jours, le débat concernant l'intérêt de la pratique d'une coelioscopie dans le seul but de découvrir une endométriose reste entier. En effet, la destruction des lésions d'endométriose stade I ou II améliore le taux de grossesses spontanées [16].

### Perspectives

Au vu de nos résultats ainsi que ceux des séries publiées, l′HSG seule parait insuffisante. La coelioscopie est un examen invasif qui comporte des risques, bien que très peu fréquents, ont conduit progressivement à abandonner sa pratique systématique. Il paraît dès lors intéressant de proposer une endoscopie aussi performante que la coelioscopie mais sans ses inconvénients. C′est pour cela que certains auteurs se sont intéressés à la fertiloscopie [17]. Il s'agit d'une endoscopie transvaginale, l'optique étant introduit dans le cul-de-sac de Douglas. Le milieu d'observation étant le sérum physiologique. Cette technique a montré son efficience en étant aussi précise que la coelioscopie permettant en outre de pratiquer en routine salpingoscopie et microsalpingoscopie [17].

## Conclusion

La coelioscopie, considérée par plusieurs auteurs comme « Gold standard » dans l'exploration du pelvis féminin et de la perméabilité tubaire en cas d'infertilité, n'est pas un examen sans failles. Les résultats de l'HSG et ceux de la coelioscopie se complètent dans cette indication. La question qui se pose donc n'est pas quel examen est juge de l'autre mais plutôt comment améliorer l'interprétation et la fiabilité de ces deux examens. L'HSG parait être très fiable lorsqu'elle est normale. Nous pensons, toutefois, qu′il existe un intérêt d′associer chaque fois que c′est possible ces deux examens. En effet, la coelioscopie permet de rattraper les fausses obstructions tubaires de l′HSG, de détecter les adhérences pelviennes ou l'endométriose. Une autre perspective pour pallier aux lacunes de l'HSG serait peut être son association à la fertiloscopie, technique encore peu diffusée, particulièrement intéressante en raison de son abord chirurgical mini-invasif.
